# The effect of processing speed on academic fluency in children with neurodevelopmental disorders

**DOI:** 10.1016/j.intell.2025.101965

**Published:** 2025-10-07

**Authors:** Soo Youn Kim, Jordyn Esprit, Ann Levine, Kevin G. Stephenson

**Affiliations:** a Child Development Center, Nationwide Children’s Hospital, 187 W. Schrock Rd., Westerville, OH 43081, USA; b Department of Pediatrics, The Ohio State University, Columbus, OH 43210, USA; c Institute for Mental and Behavioral Health Research, Nationwide Children’s Hospital, 444 Butterfly Gardens Dr., Columbus, OH 43215, USA

**Keywords:** Processing speed, Academic fluency, Neurodevelopmental disorders, Autism, ADHD, Working memory

## Abstract

Poor processing speed (PS) is frequently observed in individuals with neurodevelopmental disorders. However, mixed findings exist on the predictive validity of such processing speed impairment and the role of working memory (WM). We conducted a retrospective chart review of patients evaluated at a developmental assessment clinic between March 2018 and December 2022. Patients with available data on the Wechsler Intelligence Scale for Children, Fifth Edition (WISC-V) and the Woodcock-Johnson, Fourth Edition, Tests of Achievement (WJ IV ACH) were included (*n* = 77, 69 % male; *M*_*age*_ = 10.6, *SD*_*age*_ = 2.5; FSIQ range = 47–129). We performed a mediation analysis with academic fluency (AF) as the dependent variable, PS as the predictor, WM as the mediator, and academic skills and general intelligence as covariates. Both the direct and indirect effects of PS were significant prior to adding covariates. However, only the direct effect of PS was robust, independent of the effects of academic skills and general intelligence. The indirect effect of PS through WM was insignificant after accounting for the general academic skills and intelligence. Therefore, PS explains a unique variance in AF. This finding suggests that PS may be an exception to the criticism of cognitive profile analysis. Interpreting the PS score as a relative strength or weakness within a cognitive profile may uniquely predict their timed academic performance in youth with neurodevelopmental disorders.

Processing speed (PS) is commonly defined as the time it takes for an individual to perceive, process, and respond to a stimulus (Kail & Salthouse, 1994; Mashburn, Barnett, & Engle, 2024). While this common definition appears straightforward, the theoretical understanding of PS is more complex. On one hand, PS is regarded as a cognitive ability within the current version of the Cattell-Horn-Carroll (CHC) theory, which encompasses broad abilities related to speed, such as broad psychomotor speed, broad decision speed, broad cognitive speed, and retrieval fluency (Schneider & McGrew, 2018). On the other hand, PS is considered an independent construct comprised of multiple sub-abilities that contributes to individual differences in intelligence (Ackerman, Beier, & Boyle, 2002; Lerche et al., 2020; Mashburn et al., 2024). Diminished PS is also associated with multiple aspects of child psychopathology (e.g., Braaten et al., 2020; Kelleher, Murtagh, Clarke, Murphy, & Cannon, 2013; Mayes & Calhoun, 2007) such as depression, anxiety, psychosis, and neurodevelopmental disorders such as attention-deficit/hyperactivity disorder (ADHD) and autism spectrum disorder (ASD).

Regarding the measurement of PS, when it comes to intelligence tests, the Wechsler tests of intelligence started incorporating PS in the third edition of the Wechsler Intelligence Scales for Children (WISC-III; Wechsler, 1991). Now, all Wechsler Scales measure PS as part of their core battery (Wechsler, 2008, 2014). Namely, Coding and Symbol Search are the two core subtests included in the Wechsler Intelligence Scales for Children, fifth edition (WISC-V; Wechsler, 2014) and the Wechsler Adult Intelligence Scales, fifth edition (WAIS-5; Wechsler, 2024), the two most recent versions of the Wechsler line of intelligence tests for children and adults. The Coding subtest involves looking at a box of numbers corresponding with different symbols and copying down as many as possible in a brief period of time. The Symbol Search subtest requires test takers to determine whether symbols displayed on the left side also appear on the right. Both subtests have a time limit, so test-takers must quickly and accurately process visual information.

Despite the widespread acceptance of the Wechsler Scales, there has been continued debate on whether including PS as part of a general cognitive ability is warranted. Many sources of data support that PS may be an independent set of abilities that is less related to the Full-Scale Intelligence Quotient (FSIQ) compared to other broad ability indices. The PSI on the WISC-III, the first Wechsler test to incorporate PS, showed the lowest correlation with the FSIQ in comparison to other factors and had the lowest factor loading of 0.62 on general intelligence in contrast to other loadings of 0.85–0.90 in the standardization sample (Keith & Witta, 1997; Wechsler, 1991). This trend continued to the WISC-V, in which the PSI showed the lowest factor loading of 0.51 on FSIQ (Wechsler, 2014). More recently, a series of factor analyses on the WISC-V conducted by Canivez, Watkins, and Dombrowski (2016, 2017) following the bifactor model with partitioned higher- (i.e., general intelligence) and lower-order (i.e., broad abilities) influence on subtest variances (Cucina & Byle, 2017; Reise, 2012) demonstrated that PS was the only broad ability index that explained unique subtest variance and showed enough unique measurement based on the omega hierarchical coefficients to be interpreted independently after accounting for the influence of general intelligence. This provides additional evidence that PS is perhaps more distinct from FSIQ and the theoretical *g*-factor than other broad abilities measured by the WISC-V.

When it comes to neurodevelopmental disorders, Stephenson, Beck, South, Norris, and Butter (2021) found evidence of partial measurement invariance on the WISC-V FSIQ when comparing an autistic group to the standardization sample. Specifically, the intercepts for the Coding and Digit Span subtests were not equivalent across groups, suggesting systematic bias in assessing intelligence for autistic children. Notably, the General Ability Index, a composite that does not include either subtest, was fully invariant and was judged to be a closer approximation of general intelligence in autistic children. Reasons for the systematic differences in the Digit Span and Coding subtests, despite otherwise equivalent levels of the latent construct of IQ, were not identified. However, Stephenson and colleagues hypothesized that they may be due to broader difficulties in executive functioning skills in autistic individuals. It is robustly documented in the literature that poor PS is a frequently observed characteristic in ASD (e.g., Bucaille et al., 2016; Doobay, Foley-Nicpon, Ali, & Assouline, 2014; Velikonja, Fett, & Velthorst, 2019) and ADHD (e.g., Cook, Braaten, & Surman, 2018; Dinçer et al., 2022; Moura, Costa, & Simões, 2019). When it comes to ADHD, it is not surprising to observe PS deficits, as the condition is characterized by inattention, hyperactivity, and impulsivity. By its nature, ADHD involves deficits in several aspects of executive functions that are closely related to PS (Onandia-Hinchado, Pardo-Palenzuela, & Diaz-Orueta, 2021; Pievsky & McGrath, 2018). For ASD, although complicated due to the high co-occurrence rate with ADHD, studies have found unique associations between the diagnosis of ASD (Braaten et al., 2020) or core ASD symptoms (Oliveras-Rentas, Kenworthy, Roberson, Martin, & Wallace, 2012) and PS deficits, independent of ADHD symptoms. For example, Braaten et al. (2020) found that the relationship between PS and ASD remained after accounting for inattention, the only symptom dimension that showed significant association with slow PS. Additionally, evidence suggests that, while related, executive functioning and intelligence are separate constructs (Brydges, Reid, Fox, & Anderson, 2012).

It is well-documented that the predictive effect of broad ability IQ scores (e,g., Verbal Comprehension, Fluid Reasoning) on academic performance diminishes significantly when accounting for the general factor (e.g., Benson, Kranzler, & Floyd, 2016; Glutting, Youngstrom, Ward, Ward, & Hale, 1997, Glutting, Watkins, Konold, & McDermott, 2006; Zaboski II, Kranzler, & Gage, 2018). Given that PS is least related to FSIQ, however, it is not surprising to find mixed views on the unique predictive validity of PS. On one hand, some researchers describe the Wechsler tests’ PS subtests as “clerical” and deem the value of the subtests minimal, with very little implication beyond the ability to perform the repetitive visual-motor task (Beal, Holdnack, Saklofske, & Weiss, 2016; Schneider, 2013). Lovett, Harrison, and Armstrong (2022), in their correlational study, supported this view by concluding that PS subtests are not only, in general, a poor predictor of academic fluency (AF) or the ability to perform academic tasks accurately and quickly, but these PS subtests across different cognitive testing batteries are also highly dissimilar and thus, do not support a reliable clinical construct of PS. On the other hand, a different line of research suggests that PS is unique in its predictive effect. PS was suggested to have an important role in academic achievement, as children with slower PS were found to have poorer academic outcomes in reading and mathematics (Mayes & Calhoun, 2007; Thaler, Bello, & Etcoff, 2013).

Regarding executive functioning, Kail and Salthouse (1994) and Fry & Hale (1996) were among the first to describe the relationship between processing speed, working memory (WM) capacity, and fluid intelligence in children and young adults. These early studies found strong relations between PS, WM, and higher-order cognition, such as fluid intelligence, across the lifespan. Since then, there has been continued evidence that PS plays an important role in WM, fluid intelligence (Frischkorn, Schubert, & Hagemann, 2019; Sheppard & Vernon, 2008), and its relationship with academic achievement (McAuley & White, 2011; van der Sluis, de Jong, & ven der Leij, A., 2004). This link between PS and WM aligns with the idea that WM is time-dependent, and thus, a child with faster PS will be able to process and manipulate more relevant information before it is lost from short-term memory (Daneman & Carpenter, 1980; Weigard & Huang-Pollock, 2017).

It appears evident that there is a relationship between PS, WM, and academic achievement (Gordon, Smith-Spark, Newton, & Henry, 2018) and that diminished PS is often an observed characteristic of youth with neurodevelopmental disorders (e.g., Bucaille et al., 2016; Cook et al., 2018; Dinçer et al., 2022; Doobay et al., 2014; Moura et al., 2019; Velikonja et al., 2019). Nevertheless, there is a paucity of research investigating the predictive effect of PS on academic achievement in this population. Braaten et al. (2020) found that in youth referred for a neuropsychiatric evaluation (19 % of the sample was diagnosed with ASD and 59 % with ADHD), there was a small indirect effect of PS on an untimed reading and mathematics task via WM and a larger direct effect of PS on the untimed mathematics task after accounting for the GENERAL ABILITY INDEX. More recently, Lee (2024) found similar results with math fluency in children with ADHD. However, this study did not control for the general intelligence of the sample, which may have explained the shared variance between PS, WM, and academic achievement.

Therefore, to elucidate the predictive effect of PS on academic achievement in the neurodevelopmental population, this study aimed to examine the role of PS on AF in the context of the mediating role of WM, independent of the effect of general intelligence or basic academic skills in youth with neurodevelopmental disorders. AF was chosen as the outcome variable based on both theoretical and clinical relevance. Conceptually, AF and PS share a core emphasis on timed performance, making AF a theoretically aligned and meaningful construct to examine in relation to PS. Clinically, AF is a critical skill for academic success, as it reflects the ability to complete academic tasks both accurately and efficiently—an ability that is essential in real-world educational settings where time constraints are common (Binder, 1996; Kubina Jr. & Morrison, 2000). It was hypothesized that PS has a significant direct and indirect effect via WM on AF.

## 1. Method

### 1.1. Participants

We conducted a retrospective chart review of patients seen at a tertiary neurodevelopmental assessment clinic who were referred for various developmental and emotional/behavioral concerns at a large children’s hospital between March 2018 and December 2022. Before the evaluations, all patients completed an intake assessment to confirm concerns related to neurodevelopmental disorders. Following this, these patients completed comprehensive diagnostic evaluations, during which a wide range of potentially relevant neurodevelopmental and other psychological disorders were considered and diagnosed as clinically appropriate. We obtained a convenience sample of patients aged between 6 and 16 years with available data on the Wechsler Intelligence Scale for Children, Fifth Edition (WISC-V) Processing Speed Index (PSI) and the Woodcock-Johnson, Fourth Edition (WJ-IV) Academic Fluency (AF) and Academic Skills (AS) (*N* = 77, 69 % male; *M*_*age*_ = 10.6, *SD*_*age*_ = 2.5; FSIQ range = 47–129). There was no additional cleaning of the data or exclusion of these *N* = 77 individuals. The majority of the included patients received a diagnosis of ASD or ADHD (90 %; *n* = 69) as a result of the evaluation. Approximately half of the sample was diagnosed with ASD (46 %; *n* = 35), 71 % was diagnosed with ADHD (*n* = 55), 27 % with Language Disorder (*n* = 21), 21 % with Learning Disorder (*n* = 16), and 10 % with Intellectual Developmental Disorder (*n* = 8). See Table 1 for details on the participants’ demographic characteristics.

## 2. Measures

### 2.1. Wechsler Intelligence Scale for Children, Fifth Edition (WISC-V)

The WISC-V is the newest version of the Wechsler Intelligence Scale for Children, one of the most frequently used tests of cognitive abilities (Wechsler, 2014). The WISC-V was reported to largely reflect conceptualizations of intellectual measurement influenced by the Cattell-Horn-Carroll (CHC) theory (Wechsler, 2014). The WISC-V includes ten primary subtests that produce five factor index scores. The structural validity of the WISC-V was based on the confirmatory factor analyses (CFA) with five first-order factors (i.e., Verbal Comprehension, Visual Spatial, Fluid Reasoning, Working Memory, and Processing Speed) completely mediating the effect of general intelligence (*g*) on individual subtests (Wechsler, 2014). The WISC-V primary index scores showed good internal consistency ranging between 0.88 and 0.93 and test-retest stability across a mean interval of 26 days ranging from 0.75 to 0.94 (Wechsler, 2014). This study included the FSIQ produced by seven primary subtests, the General Ability Index (GAI) produced by five primary subtests, the Processing Speed Index (PSI) produced by two primary subtests, and the Working Memory Index (WMI) produced by two primary subtests.

### 2.2. Woodcock-Johnson, Fourth Edition, Tests of Achievement (WJ IV ACH)

The WJ IV ACH (Schrank, Mather, & McGrew, 2014) is one of the most popular individually administered tests of academic achievement among school and clinical psychologists. It is part of the three WJ IV batteries, designed to reflect several CHC factors (McGrew, LaForte, & Schrank, 2014). The WJ IV ACH produces two total achievement scores (i.e., broad and brief) and 19 cluster scores, including Broad Reading, Basic Reading Skills, Broad Mathematics, Broad Written Language, Academic Skills, and Academic Fluency. The WJ IV ACH showed good internal consistency for non-speeded composites (i.e., median *r* = 0.89–0.96 across age) and good test-retest reliability in speeded tasks across a 1-day interval (i.e., *r* = 0.76–0.97; McGrew et al., 2014). In this study, two cluster scores, Academic Fluency and Academic Skills, were used. Academic Fluency consists of three subtests: Sentence Reading Fluency, Math Facts Fluency, and Sentence Writing Fluency. Academic Skills includes three subtests: Letter-Word Identification, Spelling, and Calculation.

## 3. Statistical analysis

We used Pearson correlations to summarize the relationships between our included variables. A mediation analysis was performed using the PROCESS macro (Hayes, 2022), which involves a series of multiple regression analyses. The WJ IV ACH Academic Fluency (AF) was specified as the dependent variable, the WISC-V PSI as the predictor, and the Working Memory Index (WMI) as the mediator. The WJ IV ACH Academic Skills and the WISC-V General Ability Index were included as covariates. All variables included in the mediation analysis were chosen a priori. All analyses were done using IBM SPSS Statistics (Version 28). No data were missing from any of the included variables.

## 4. Results

First, the statistical assumptions for path analysis were evaluated. The Kolmogorov–Smirnov test confirmed that residuals met the assumption of normality, with *D* = 0.095 and *p* = .079. Visual inspection of the Q-Q plot also suggested that the residuals were approximately normally distributed. Furthermore, predictors exhibited tolerance values from 0.50 to 0.71 and Variance Inflation Factor (VIF) scores of 1.40 to 2.14, suggesting that multicollinearity was not a concern in the current regression analysis.

**Fig. 1.**
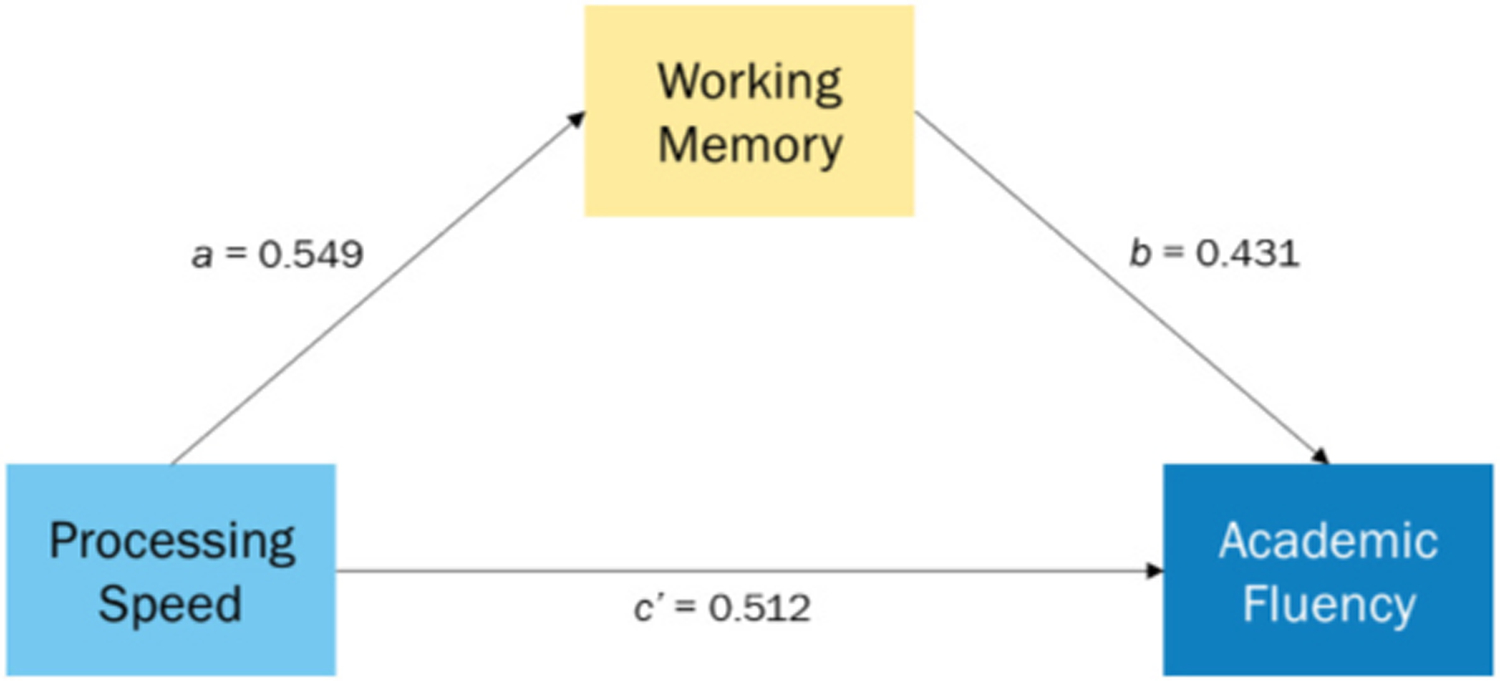
Results of simple mediation analysis.

A correlational analysis was initially performed to explore the relationships among variables (See Table 2). From a mediation analysis conducted using ordinary least square path analysis, PSI showed both a direct effect on AF and an indirect effect through WMI. As seen in Fig. 1, youth with higher scores on the PSI also showed higher scores on the WMI (*a* = 0.549, *p* < .0001), and youth with higher scores on the WMI showed higher scores on AF (*b* = 0.431, *p* < .0001). A 95 % bootstrap confidence interval for the indirect effect (*ab* = 0.237, 32 % of the total effect) based on 10,000 bootstrap samples was entirely above zero ([0.113, 0.370]). Independent of its indirect effect, PSI also had a direct effect on AF (*c’* = 0.512, *p* < .0001).

**Fig. 2.**
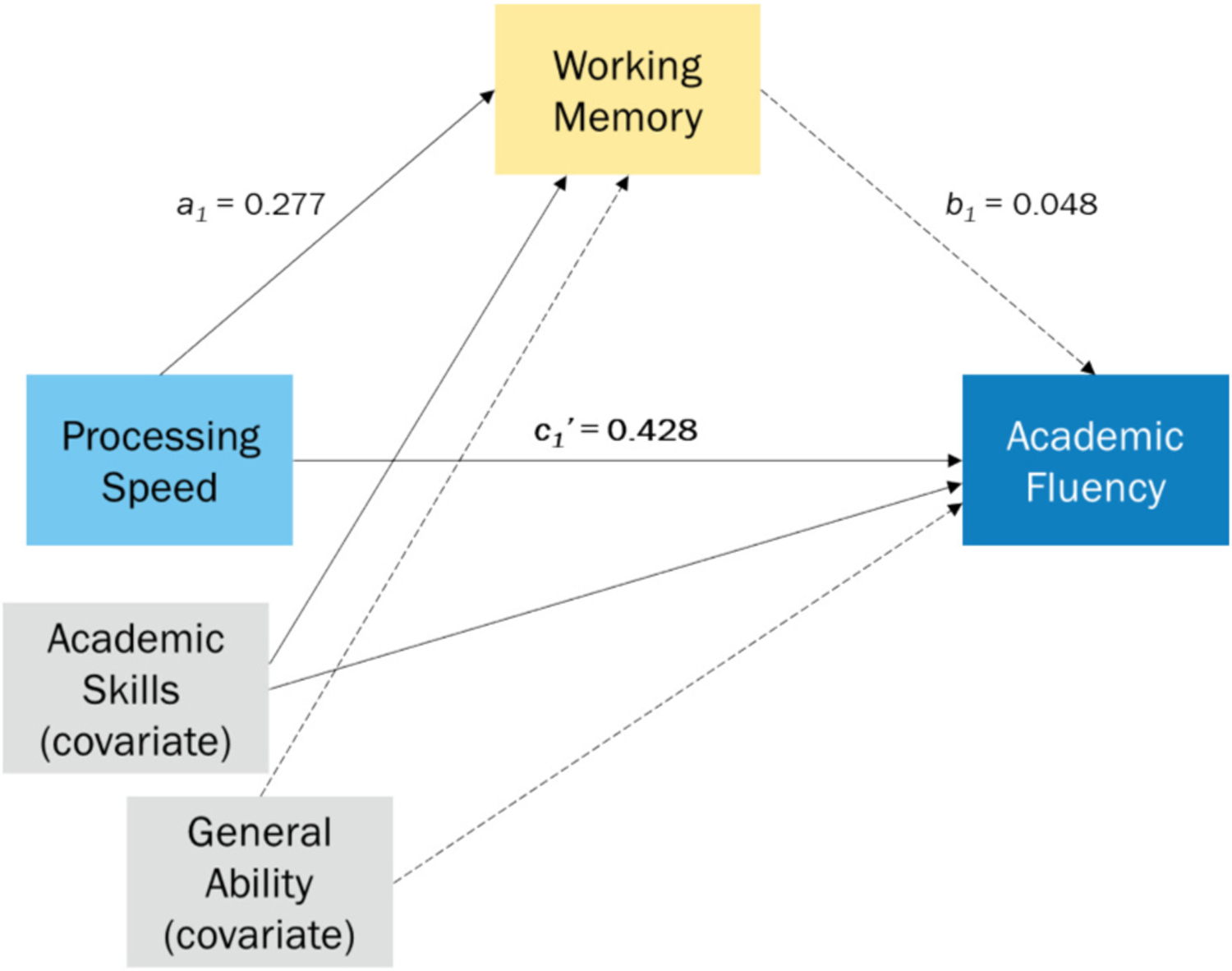
Mediation analysis controlling for Academic Skills and General Cognitive Ability. *Note*. Solid line indicates a statistically significant relationship and dashed line indicates a statistically nonsignificant relationship.

However, when Academic Skills and the General Ability Index were included in the mediation analysis as covariates, as seen in Fig. 2, the indirect effect of PSI through WMI was eliminated (*ab* = 0.013; 95 % bootstrap confidence interval [−0.025, 0.068]). The direct effect of PSI on AF continued to be robust (*c*_*1*_*’* = 0.428, *p* < .0001). Academic Skills was also a significant predictor of WMI (*a*_*2*_ = 0.416, *p* < .0001) and AF (*c*_*2*_*’* = 0.542, *p* < .0001). The General Ability Index did not significantly predict either WMI (*a*_*3*_ = 0.154, *p* = .068) or AF (*c*_*3*_*’* = 0.044, *p* = .436). Refer to Table 3 for statistical details on the mediation analyses.

## 5. Discussion

This study aimed to examine the predictive validity of PS on AF in children with various neurodevelopmental disorders. Both the direct and indirect effects of PS on AF were significant prior to adding covariates, with a substantially larger direct effect. However, after controlling for the effects of Academic Skills and the General Ability Index, the mediating effect of WM on AF was no longer significant. The direct effect of PS on AF was robust, independent of the effects of general academic skills and intellectual abilities on shared variances between the relationships of PS, WM, and AF. This highlights the unique predictive validity of PS on AF, which is the ability to perform academic tasks accurately and quickly. In other words, the utilization of WM is minimal when it comes to simple, timed academic tasks after accounting for other cognitive processes (i.e., PS, Academic Skills, General Ability Index). The relationship between WM, PS, and academic achievement has been well-documented (Gordon et al., 2018). However, only a few recent studies examined the relationship between PS, WM, and AF in youth with neurodevelopmental disorders (Braaten et al., 2020; Lee, 2024). Although some of our findings replicated what has been found in previous literature (Braaten et al., 2020; Gordon et al., 2018; Lee, 2024), our findings are unique in that PS showed a direct but no indirect effect via WM on AF. This difference in finding may potentially be due to the use of timed compared to untimed tasks (Braaten et al., 2020) and the inclusion of covariates to control for the general academic and intellectual abilities (Lee, 2024). It is important to note that Academic Skills (but not the General Ability Index) was found to be a significant covariate explaining the relationship between PS and WM and WM and AF.

Cognitive profile analysis has been criticized due to its lack of psychometric support, including poor temporal stability of broad ability scores (e.g., Watkins & Canivez, 2004; Watkins & Smith, 2013), the diminished predictive effect of broad ability indices when accounted for general intelligence (e.g., Benson et al., 2016; Glutting et al., 1997; Glutting et al., 2006; Zaboski II et al., 2018), and lack of evidence in conveying useful clinical information (e.g., Macmann & Barnett, 1997; Smith & Watkins, 2004; Watkins, Kush, & Schaefer, 2002). Contrary to these findings, the current study provides novel information that PS could be a unique ability with a predictive utility that differentiates itself from other broad abilities, especially when considering the existing research on how the PSI performs differently with high unique explained variance and low factor loadings on the FSIQ (Canivez et al., 2016, 2017; Keith & Witta, 1997; Wechsler, 1991, 2014). This leads to important practical implications, as the WISC-V PSI is often used in cognitive profile analysis (e.g., Pattern of Strengths and Weaknesses approach; Flanagan et al., 2018) and in clinical settings to create recommendations for academic accommodations, such as extended time and shortened assignments (Gordon et al., 2018). In the present study, as expected, children with higher functioning on the PS, WM, Academic Skills, or the General Ability Index tended to show higher scores on AF. However, regarding distinct contributions, PS and Academic Skills played unparalleled roles in AF. Although this study did not assess for the specificity of this effect of PS, given previous studies on the limited predictability of IQ indices (e.g., Benson et al., 2016; Glutting et al., 1997, 2006; Zaboski II et al., 2018), it is most likely that the effect of PS is specific to AF and no other academic outcomes.

WM is undoubtedly an important executive function that plays a significant role in many academic achievements, including reading comprehension and mathematic problem-solving (Borella & de Ribaupierre, 2014; Caviola, Mammarella, Cornoldi, & Lucangeli, 2012; Rasmussen & Bisanz, 2005). Despite its importance, we found that WM may account for a minor role in AF in this population, after accounting for PS and AS. In fact, Academic Skills explained a substantial portion of the shared variance between WM and AF, indicating that for discrete speed-based academic tasks, basic academic skills and mental speed are two crucial components. Similarly, although the predictive validity of general intelligence and academic achievement is well-established (Deary, Strand, Smith, & Fernandes, 2007; Giofrè, Borella, & Mammarella, 2017), our study surprisingly found that the General Ability Index was not a significant covariate in the relationship between PS and AF once Academic Skills was included. The WJ IV ACH Academic Skills consists of three subtests focused on core academic principles: Letter-Word Identification, Spelling, and Calculation. Given that the WJ IV ACH AF subtests are also based on simple tasks such as sentence reading, sentence writing, and basic calculation, the specific focus on academic principles may explain the larger shared variance with Academic Skills but not the General Ability Index.

From a different perspective, the finding that Academic Skills, but not the General Ability Index, was a significant covariate may also suggest that AF is a skill related to, yet conceptually distinct from, general academic achievement. At its core, AF is the combination of accuracy and speed that leads to competent performance (Binder, 1996; Kubina Jr. & Morrison, 2000). AF is crucial for students in real school settings. AF allows students to complete tasks on time and to efficiently utilize their cognitive resources for higher-level thinking and comprehension (Álvarez-Cañizo, Suárez-Coalla, & Cuetos, 2015; Bigozzi, Tarchi, Vagnoli, Valente, & Pinto, 2017). Given that children with neurodevelopmental disorders often have impairments in PS (e.g., Bucaille et al., 2016; Cook et al., 2018; Dinçer et al., 2022; Doobay et al., 2014; Moura et al., 2019; Velikonja et al., 2019) and AF (Benallie et al., 2021), this finding leads to important implications for selecting interventions and outcomes. Although there are studies on the effectiveness of interventions on AF (e.g., Hudson, Koh, Moore, & Binks-Cantrell, 2020; Stevens, Walker, & Vaughn, 2017; Stocker Jr, Schwartz, Kubina Jr, Kostewicz, & Kozloff, 2019), research on interventions for PS has largely shown to have a task-specific but not meaningful generalized effect (e.g., Norton, 2020). It is important for future studies to explore whether AF and PS have a bidirectional relationship and, therefore, whether remedying deficits in AF would have an effect on PS. More research is essential to clarify how a low PS is clinically defined (e.g., a meaningful difference between PS and other indices), the potential utility of a profile analysis centered on PS, and the appropriate type and intensity of supports and compensatory strategies that can remediate PS deficits in an academic setting.

## 6. Limitations

The current results should be interpreted in light of several limitations. First, this study was conducted through a retrospective chart review, which entails limitations such as potential selection bias (i.e., only individuals referred for clinical evaluation) and reliance on the data available in medical records, limiting access to variables like socioeconomic information. Nevertheless, the advantages of a retrospective chart review study, when done meticulously with necessary considerations, such as clearly conceptualizing the research question, are well-documented (Gearing, Mian, Barber, & Ickowicz, 2006; Vassar & Holzmann, 2013). Second, the WISC-V PSI and the WJ IV ACH AF subtests require graphomotor abilities. There is a possibility that individuals’ fine motor ability, which is not measured and included in this study, is the shared factor between PS and AF. We encourage researchers in future studies to assess PS using multiple modalities such as graphomotor, visual matching, and oral tasks from the WISC-V Integrated (Wechsler & Kaplan, 2015) and the NIH Toolbox (Toolbox Assessments, Inc, 2023) to decrease the reliance on fine motor skills. Third, this study employed a mediation analysis that inherently assumes causality using cross-sectional data. While results should be interpreted with caution, considering the temporal stability of cognitive functions (Breit, Scherrer, Tucker-Drob, & Preckel, 2024) and the theoretical support that early cognitive and executive functioning predicts academic achievement (e. g., Bornstein, Hahn, & Wolke, 2013; Robson, Allen, & Howard, 2020; Zelazo & Carlson, 2020), we can reasonably assert that the current findings accurately represent the relationship between PS and AF. Future research should adopt a longitudinal approach to examine causality effectively. Additionally, we did not consider other academic outcomes that are not time-based, such as reading comprehension, math problem-solving, and written expression, nor have we included any executive functions apart from WM. Therefore, we could not determine whether the effect of PS is specific to AF or if its impact is mediated by different executive functions. Future research should expand its scope to investigate how a broader range of executive, cognitive, and academic functions are related.

## 7. Conclusions

The current study examined the relationship between PS, WM, and AF. Results showed that PS has a strong direct predictive effect on AF even after controlling for the General Ability Index and AS. It is important to note that Academic Skills, but not the General Ability Index, significantly contributed to predicting AF. Such findings suggest that PS uniquely predicts AF and that both PS and Academic Skills are foundational for success in discrete, speed-based academic tasks. Processing speed may serve as a valuable marker for guiding future research aimed at identifying effective strategies to support children with low academic fluency.

## Figures and Tables

**Table 1:** Participant Characteristics (*N* = 77).

Age (years)	
Mean (SD)	10.6 (2.5)
Median [Min, Max]	10 [6, 16]
Race	
Bi-racial/Multi-racial	5 (6.5 %)
Black	7 (9.1 %)
Unknown	3 (3.9 %)
White	62 (80.5 %)
Biological Sex	
Female	24 (31.2 %)
Male	53 (68.8 %)
Neurodevelopmental Diagnosis	
Autism Spectrum Disorder	35 (46 %)
Attention-Deficit/Hyperactivity Disorder	55 (71 %)
Intellectual Developmental Disorder	8 (10 %)
Any Language Disorder	21 (27 %)
Any Learning Disorder	16 (21 %)
WISC-V FSIQ	
Mean (SD)	88.5 (15.3)
Median [Min, Max]	88 [47, 129]
WISC-V GAI	
Mean (SD)	89.2 (17.4)
Median [Min, Max]	89 [47, 133]
WISC-V PSI	
Mean (SD)	87.0 (14.5)
Median [Min, Max]	89 [45, 114]
WISC-V WMI	
Mean (SD)	90.2 (15.2)
Median [Min, Max]	91 [59, 130]
WJ IV ACH Academic Fluency	
Mean (SD)	85.5 (15.6)
Median [Min, Max]	85 [48, 116]
WJ IV ACH Academic Skills	
Mean (SD)	88.6 (16.8)
Median [Min, Max]	92 [41, 132]

*Note*. WISC-V = Wechsler Intelligence Scale for Children, Fifth Edition, FSIQ = Full Scale Intelligence Quotient, GAI = General Ability Index, PSI = Processing Speed Index, WMI = Working Memory Index, WJ IV ACH = Woodcock-Johnson, Fourth Edition, Tests of Achievement.

**Table 2:** Pearson correlation coefficients among cognitive and academic variables.

	PSI	WMI	GAI	AF	AS
PSI	1.00				
WMI	0.52***	1.00			
GAI	0.32**	0.50***	1.00		
AF	0.69***	0.67***	0.51***	1.00	
AS	0.44***	0.67***	0.53***	0.81***	1.00

*Note*. WMI = WISC-V Working Memory Index, PSI = WISC-V Processing Speed Index, GAI = WISC-V General Ability Index, AF = WJ ACH IV Academic Fluency, AS = WJ ACH IV Academic Skills.

** *p* < .01.

*** *p* < .001.

**Table 3:** Regression statistics completed as part of the mediation analyses.

Predictors	Estimate	*SE*	*t*	*p*	95 % CI
	Outcome: WMI				
(Intercept)	42.47	9.16	4.64	< 0.0001	[24.22, 60.72]
PSI	0.55	0.10	5.28	< 0.0001	[0.34, 0.76]
*R^2^* = 0.271					
	Outcome: AF				
(Intercept)	2.01	7.86	0.26	0.799	[−13.65, 17.67]
PSI	0.51	0.09	5.56	< 0.0001	[0.33, 0.69]
WMI	0.43	0.09	4.94	< 0.0001	[0.26, 0.61]
*R^2^* = 0.609					
	Outcome: AF				
(Intercept)	20.33	7.94	2.56	0.012	[4.52, 36.14]
PSI	0.75	0.09	8.32	< 0.0001	[0.57, 0.93]
*R^2^* = 0.480					
	Outcome: WMI				
(Intercept)	15.53	8.72	1.78	0.079	[−1.84, 32.91]
PSI	0.28	0.09	2.93	0.005	[0.09, 0.47]
GAI	0.15	0.08	1.85	0.068	[−0.01, 0.32]
AS	0.42	0.09	4.59	< 0.0001	[0.24, 0.60]
*R^2^* = 0.532					
	Outcome: AF				
(Intercept)	−8.06	5.92	−1.36	0.178	[−19.86, 3.74]
PSI	0.43	0.07	6.45	< 0.0001	[0.30, 0.56]
WMI	0.05	0.08	0.61	0.542	[−0.11, 0.20]
GAI	0.04	0.06	0.78	0.436	[−0.07, 0.16]
AS	0.54	0.07	7.92	< 0.0001	[0.41, 0.68]
*R^2^* = 0.806					
	Outcome: AF				
(Intercept)	−7.32	5.77	−1.27	0.209	[−18.82, 4.18]
PSI	0.44	0.06	7.06	< 0.0001	[0.32, 0.57]
GAI	0.05	0.06	0.94	0.351	[−0.06, 0.16]
AS	0.56	0.06	9.36	< 0.0001	[0.44, 0.68]
*R^2^* = 0.805					

*Note*. WMI = WISC-V Working Memory Index, PSI = WISC-V Processing Speed Index, GAI = WISC-V General Ability Index, AF = WJ ACH IV Academic Fluency, AS = WJ ACH IV Academic Skills.

## Data Availability

The authors do not have permission to share data.
